# Early Interleukin-22 and Neutrophil Proteins Are Correlated to Future Lung Damage in Children With Cystic Fibrosis

**DOI:** 10.3389/fped.2021.640184

**Published:** 2021-03-31

**Authors:** Julie Renwick, Emma Reece, Jamie Walsh, Ross Walsh, Thara Persaud, Cathal O'Leary, Seamas C. Donnelly, Peter Greally

**Affiliations:** ^1^Clinical Microbiology, Trinity College Dublin, Dublin, Ireland; ^2^Children's Health Ireland and Tallaght University Hospital, Dublin, Ireland; ^3^Department of Medicine, Tallaght University Hospital and Trinity College Dublin, Dublin, Ireland

**Keywords:** cystic fibrosis, proteomics, bronchoalveolar lavage, neutrophils, biomarkers

## Abstract

Cystic Fibrosis (CF) lung damage begins early in life. Lung function decline is associated with pulmonary infections, neutrophil infiltration and inflammation. In CF, neutrophils have an altered phenotype. In this pilot study, we aimed to determine if signals of dysfunctional neutrophil responses were evident early in life and whether these signals may be associated with lung damage in later childhood. We examined the pulmonary protein profiles of 14 clinical stable infants and pre-school children with CF employing the aptamer-based affinity platform, SOMAscan®. High resolution computed tomography (HRCT) was performed on all children after age 6 years and Brody score calculated. A Spearman's rank order correlation analysis and Benjamini-Hochberg adjustment was used to correlate protein concentrations in early life to Brody scores in later childhood. Early life concentrations of azurocidin and myeloperoxidase, were positively correlated with Brody score after age 6 (*p* = 0.0041 and *p* = 0.0182, respectively). Four other neutrophil associated proteins; Complement C3 (*p* = 0.0026), X-ray repair CCP 6 (*p* = 0.0059), C3a anaphylatoxin des Arginine (*p* = 0.0129) and cytokine receptor common subunit gamma (*p* = 0.0214) were all negatively correlated with Brody scores. Interestingly, patients with more severe lung damage after age 6 had significantly lower levels of IL-22 in early years of life (*p* = 0.0243). IL-22 has scarcely been reported to have implications in CF. Identification of early biomarkers that may predict more severe disease progression is particularly important for the future development of early therapeutic interventions in CF disease. We recommend further corroboration of these findings in prospective validation studies.

## Introduction

Cystic fibrosis (CF) is characterized by frequent pulmonary exacerbations resulting in bronchiectasis, irreversible lung damage and eventually lung failure. The path of disease progression is established early in life with lung damage already evident in patients with CF as early as 6 years of age (1). Bacterial infections and the associated inflammation are the most common cause of morbidity and lung function decline (2). One of the clinical hallmarks of CF is the increased burden of neutrophils in the airways. Neutrophils are not only important effectors of bacterial phagocytosis, but they also dominate the inflammatory response. In the CF airways, neutrophils show a dysfunctional phenotype that deviates from their wild type counterparts (3).

High resolution computed tomography (HRCT) is accepted as a sensitive means of detecting early structural lung disease in CF, demonstrating abnormalities before the onset of clinical, spirometric, or plain radiographic abnormalities (4, 5). HRCT scanning is widely available and validated scoring systems have been developed (5, 6). Brody et al. (7) developed a sensitive, reproducible HRCT scoring system to evaluate CT features of CF lung disease suitable for use in children. Images are assessed for the lobar location and severity of morphological and parenchymal changes. These include bronchiectasis, peri-bronchial thickening, mucous plugging, atelectasis, consolidation, air/fluid levels, hyperinflation, and overall impression (6, 8).

We hypothesize that evidence of heightened neutrophil degranulation within the lungs of CF patients early in life will correlate with enhanced pulmonary injury later in life. Here we examined pulmonary proteins from bronchoalveolar lavage (BAL) in the first years of life that may be predictive of CF disease severity later in life. In CF, signals of early neutrophil dysfunction could be used to identify patients at risk of following a more severe disease pathway. We therefore were specifically interested in defining the presence or absence of specific proteins that have been implicated in neutrophil biological activity. This study aimed to identify BAL fluid proteins present in early life that correlate with severity of CF lung disease after age 6.

## Materials and Methods

BAL was obtained from 14 clinically stable infants and pre-school children (median age 2 years, range 1–5 years) with CF undergoing bronchoscopy as part of an annual surveillance program (Table 1). Clinical stability was defined as being asymptomatic with no change from baseline for 4 weeks prior to BAL. Bronchoscopy was performed through a laryngeal mask airway instilling 1 ml/kg sterile 0.9% NaCl per aliquot twice in the right middle lobe and twice in the lingula. Pooled aliquots were centrifuged at 10,000 × *g* for 10 min. The protein content of supernatants was determined by Quant-iT^TM^ protein assay kit (Thermo Fisher). A minimum concentration of 20/200 μl of each sample was shipped to SOMAscan for proteomic analysis. SOMAscan® is an aptamer-based affinity platform capable of measuring 1,305 human protein analytes in lung fluid with high sensitivity and specificity (9). High resolution computed tomography (HRCT), free breathing on inspiration was performed on all children during clinical stability at their most recent clinic (median age 6.5 years, range 5–10 years) and Brody score calculated by blinded radiologist (7). The Brody lobular score system has been shown to be a sensitive method for evaluating the progression of CF lung disease (6). There was a median of 4.5 years between initial BAL and follow-up HRCT (range 1–7 years). All statistical analyzes were performed using GraphPad PRISM version 8. *P* < 0.05 were considered statistically significant. To correlate protein concentration to Brody scores, a Spearman's rank order correlation analysis and Benjamini-Hochberg adjustment with a false discovery rate of *q* = 0.10 was performed.

**Table 1:** Patient demographics and clinical details at time of BAL collection.

	**Male**	**Female**
Patient number (*n*, %)	7 (50)	7 (50)
Age (years) (mean, +/– SD)	2.1 (0.9)	2.5 (1.5)
BMI z score (median, +/– SD)	0 (0.89)	0 (0.82)
F508del/F508del (*n*, %)	3 (42.9)	3 (42.9)
F508del/G551D (*n*, %)	1 (14.2)	1 (14.2)
F508del/other (*n*, %)	3 (42.9)	3 (42.9)
Ivacaftor (*n*, %)	1 (14.3)	0 (0)
Long-term prophylactic antibiotics (*n*, %)	4 (57.1)	2 (28.6)
*P. aeruginosa* positive patients (*n*, %)	2 (28.6)	1 (14.3)
*S. aureus* positive patients (*n*, %)	5 (71.4)	4 (57.1)

*BMI, body mass index; n, number; SD, standard deviation*.

*No patients were on Lumacaftor, prednisolone, antifungals, or inhaled antibiotics at the time of BAL collection*.

## Results

**Figure 1:**
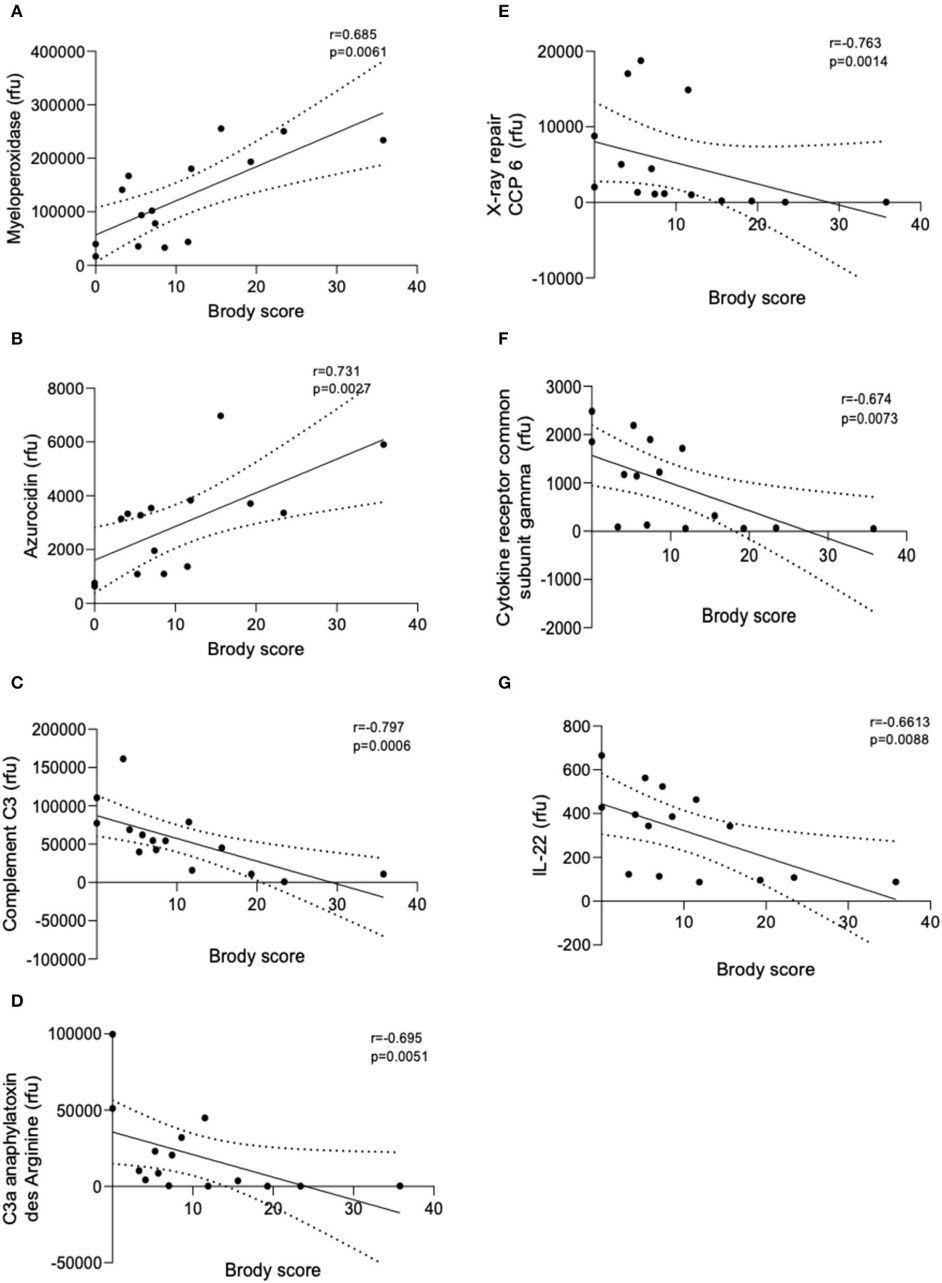
Linear regression models of BAL protein concentrations correlated with Brody score. Linear regression analysis of the relative fluorescent units (rfu) of the seven proteins that correlated with Brody scores. **(A)** Myeloperoxidase, **(B)** Azurocidin, **(C)** Complement C3, **(D)** C3a anaphylatoxin des Arginine, **(E)** X-ray repair CCP-6, **(F)** Cytokine receptor common subunit gamma, and **(G)** IL-22. Spearman's Rho = r. Coefficient of determination = *R*^2^. Dashed lines represent 95% confidence intervals.

SomaScan detected a total of 234 proteins and of these 18 were significantly correlated with Brody Scores. All proteins were detected at or above the limit of detection (LOD) of 38rfu (SomaScan white sheet). A total of 7 proteins implicated in inflammation and neutrophil function were observed to correlate with Brody scores (Figure 1). Early life concentrations of azurocidin and myeloperoxidase, (two neutrophil granule proteins) were significantly higher in patients with greater lung damage after age 6 (*p* = 0.0041 and *p* = 0.0182, respectively). Four immune proteins; Complement C3 (*p* = 0.0026), X-ray repair CCP 6 (*p* = 0.0059), C3a anaphylatoxin des Arginine (*p* = 0.0129) and cytokine receptor common subunit gamma (*p* = 0.0214) were all negatively correlated with Brody scores. Patients with more severe lung damage after age 6 had significantly lower levels of IL-22 in early years of life (*p* = 0.0243).

## Discussion

Sputum biomarkers of infection and inflammation have previously been suggested as correlates of present disease severity (10). In particular elastase, interleukin-8 (IL-8), matrix metalloproteinase (MMP)-9 and neutrophil counts were negatively correlated with current lung function (10, 11). Neutrophil elastase activity in BAL fluid in early life was associated with early bronchiectasis in children with CF (12).

Azurocidin is an antibacterial glycoprotein released from neutrophils upon degranulation in response to tissue injury or infection. Azurocidin recruits and activates monocytes resulting in cytokine release and increased phagocytosis. Myeloperoxidase is a peroxidase enzyme that catalyzes the formation of reactive oxygen intermediates which play an important role in microbial killing. Myeloperoxidase, present in the lung fluid of CF patients, has been suggested to contribute to lung tissue destruction and the early pathogenesis of CF (13). Both of these neutrophil granule proteins were correlated to more severe lung damage after age 6 suggesting early disruptions in neutrophil function and excessive neutrophil degranulation as determinants of later disease severity. Of note, IL-8 and neutrophil elastase were not correlated to Brody score in this study however this may be due to the young age of patients and the relative stability at time of bronchoscopy.

Interleukin-22 (IL-22) is involved in mucosal host defenses including tissue repair and protection, increasing innate defenses and maintaining epithelial barrier functions. IL-22 has been shown to provide critical immunity against a number of pathogens including *Klebsiella pneumoniae, Streptococcus pneumoniae, Pseudomonas aeruginosa*, and *Aspergillus fumigatus* (14). It has been suggested that the presence of neutrophils in the lungs of CF patients results in an acquired deficiency of the IL-22 pathway due to IL-22 degradation by neutrophil proteases (15). IL-22 has also been negatively correlated with neutrophil recruitment in the lungs (16). Here we observed that low levels of IL-22 in early life were correlated with more severe lung damage after age 6. Low levels of IL-22 early in life may pre-dispose certain sub-populations of CF patients to poorer immunological responses to infection, increased neutrophil recruitment and so contribute to greater lung damage later in life. Complement C3, C3a, X ray repair CCP6, and common receptor cytokine subunit gamma were all negatively correlated with Brody scores. The complement component C3 plays a central role in the activation of the complement system. Derived from C3, C3a anaphylatoxin is a potent local inflammatory mediator. Xray repair CCP6 is a DNA repair protein and the common receptor cytokine submit gamma is integral to regulating immune system functions. Low levels of these proteins may lead to dysfunctional immune responses. Nonetheless, this study has a number of limitations. The main limitation of this study is its small sample size. The widely used aptamer-based assay, SomaScan, provides relative protein quantification and further studies should include absolute quantification methods in combination. Considering these limitations results should be interpreted with caution and larger validation studies should be conducted to corroborate these exciting findings.

## Conclusion

Identification of early biomarkers is particularly important in order to intervene in the progression of CF disease. We have shown that several neutrophil proteins in early CF BAL correlate with more severe lung disease later in childhood and therefore may be useful in identifying particularly vulnerable patient populations. Additionally, IL-22 may be a novel target to investigate in the inflammatory process in CF. We recommend further corroboration of these findings in prospective validation studies. This is the first description of early life IL-22 levels correlating with worse CF lung injury in later life.

## Data Availability Statement

All Data relevant to this study is displayed in Table 1.

## Ethics Statement

The ethics for this study was reviewed and approved by Tallaght University Hospital & St James's Hospital joint research ethics committee (2019-09 List 35(01)). Written informed consent to participate in this study was provided by the participants' legal guardian/next of kin.

## Author Contributions

JR, SD, and PG: conception of study. PG: performed bronchoscopies and collected samples. JR and ER: carried out laboratory work. CO'L, JR, ER, JW, and RW: collated clinical data and analyzed data. TP: performed brody scoring. JR, ER, SD, and PG: wrote paper. All authors read and approved the final manuscript.

## Conflict of Interest

The authors declare that the research was conducted in the absence of any commercial or financial relationships that could be construed as a potential conflict of interest.

## Abbreviations

CF: cystic fibrosis
BAL: bronchoalveolar lavage
IL: interleukin
MMP-9: matrix metalloproteinase 9.
